# Tracking cellular and molecular changes in a species-specific manner during experimental tumor progression *in vivo*

**DOI:** 10.18632/oncotarget.24598

**Published:** 2018-03-01

**Authors:** Emilie Indersie, Katarzyna B. Hooks, Caroline Capdevielle, Monique Fabre, Nathalie Dugot-Senant, Angélique Desplat, Sébastien Lepreux, Aksam Merched, Christophe F. Grosset, Martin Hagedorn

**Affiliations:** ^1^ University Bordeaux, INSERM U1035, miRCaDe team, Biothérapie des Maladies Génétiques, Inflammatoires et du Cancer, Bordeaux 33076, France; ^2^ University Bordeaux, INSERM U1035, Bordeaux Research in Translational Oncology (Bariton), Bordeaux 33076, France; ^3^ Necker Hospital, Paris 75015, France; ^4^ Plateforme d’histologie UMS 005, Bordeaux 33000, France; ^5^ Service de Pathologie, CHU de Bordeaux, Bordeaux 33000, France

**Keywords:** hepatoblastoma, RNA-sequencing, chick chorioallantoic membrane, bioinformatics, experimental tumor model

## Abstract

Hepatoblastoma (HBL) is a pediatric liver cancer with defined molecular alterations driving its progression. Here, we describe an animal model for HBL on the chick chorioallantoic membrane (CAM), which recapitulates relevant features of HBL in patients. Expression of classic tumor-associated proteins such as β-catenin, EpCAM and CK19 was maintained in acini-like organized tumors on CAM, as was synthesis of AFP, a tumor marker used for monitoring patient response. RNA sequencing revealed an unexpected molecular evolution of HBL cells on the CAM, with significant deregulation of more than 6,000 genes including more than half of all HOX genes. Bioinformatic analysis distinguish between tumor cell-expressed genes and chick genes, thereby shedding new light on the complex interactions taking place during HBL progression. Importantly, human tumor suppressive ribosomal genes were downregulated after implantation, whereas mitochondrial genes encoding for anti-apoptotic peptides were strongly induced *in vivo*. Meprin-1α expression was increased during evolution of CAM tumors and confirmed by immunohistochemistry. Cisplatin, a commonly used chemotherapeutic agent for HBL, showed significant anti-tumoral effects. Our results broaden the understanding of the molecular adaptation process of human cancer cells to the microenvironment and might help to elaborate novel therapeutic concepts for the treatment of this pediatric liver tumor.

## INTRODUCTION

Tumor progression depends on molecular interactions of malignant cells with their surrounding stroma. After initiation of tumor development, dividing cancer cells need to establish communication with normal cells in order to receive nutrients and adhesion. *In vivo* modeling of these basic aspects of malignant tumor growth is important to identify novel therapeutic targets or biomarkers. There are only few reports about *in vivo* models of hepatoblastoma (HBL), [1, 2] even though the Huh6 cell line has been established more than 40 years ago. There is therefore a need for novel cell lines and models for HBL [3]. HBL, the most common pediatric liver cancer, is classified into histological subtypes reflecting different stages of liver development [4]. Etiology of the disease is unclear with few validated information available, but low birth weight and tobacco use of parents are emerging as factors favoring HBL [5]. Treatment options for HBL include chemotherapy, surgery or liver transplantation resulting in a five-year survival up to 85% [6]. Pretreatment staging and treatment outcome are defined by international collaborative research consortiums such as PRETEXT (PRETreatment EXTent of disease) and CHIC (Children’s Hepatic Tumors International Collaboration) [7]. In general, HBL respond well to chemotherapy. However, some tumors are resistant and chemotherapy can have dramatic effects on developing children, such as ototoxicity and severe hematological complications [8]. Therefore, novel therapeutic approaches and targets are needed to overcome limitations of current treatments.

On a molecular level, deregulations of several important signaling pathways have been evidenced in HBL. Signaling networks active during normal development become deregulated in HBL, including sonic hedgehog, MYC and Wnt, [9, 10] the latter being mainly triggered by gain-of-function mutations in the *CTNNB1* gene [11, 12]. These molecular alterations, which are thought to drive tumor initiation and progression, might become druggable targets in the future, once potent inhibitors are identified and validated. We have recently shown that microRNAs targeting beta-catenin might be used to inhibit HBL growth *in vitro* and *in vivo*, [13] opening the way to target oncogenes which are difficult to block pharmacologically. Previously, microarray analyses of HBL tumors have led to the identification of a 16-gene signature, which distinguishes between high risk and low risk patients [14]. Expression of a few genes can thus be informative for clinical outcome in HBL.

The chick embryo has been used successfully to gain insight into the interactions of malignant human cells of various origins with stromal cells of the host with a particular emphasis on lymphatic and blood vascular cells, including action of tumor-secreted growth factors such as VEGF [15–18]. The combination of a pertinent *in vivo* model with modern high-throughput microarrays allows discovery of molecular mechanisms driving tumor progression [16, 19]. Cross-species hybridization of experimental tumors grown on the CAM simultaneously on human and chick gene chips has broadened our understanding of tumor-stroma interaction, because tumor-cell regulated genes can be distinguished from stroma-expressed genes. Using this strategy *NTN1* and *CXCL4V1* have been identified as key tumor-promoting factors produced in pancreatic tumor cells [19, 20].

Here we used high throughput RNA sequencing (RNA-seq) to decipher molecular changes occurring during adaptation of HBL-derived Huh6 cells from *in vitro* growth condition to *in vivo* evolution on the CAM, separating tumor-cell gene regulation from the stroma compartment. Several genes identified in this model have never been described in the context of HBL. Their potential role for hepatoblastoma growth is discussed and their contribution to HBL progression warrants further investigation.

## RESULTS

### Morphological characterization of Huh6 cell growth on the CAM

**Figure 1:**
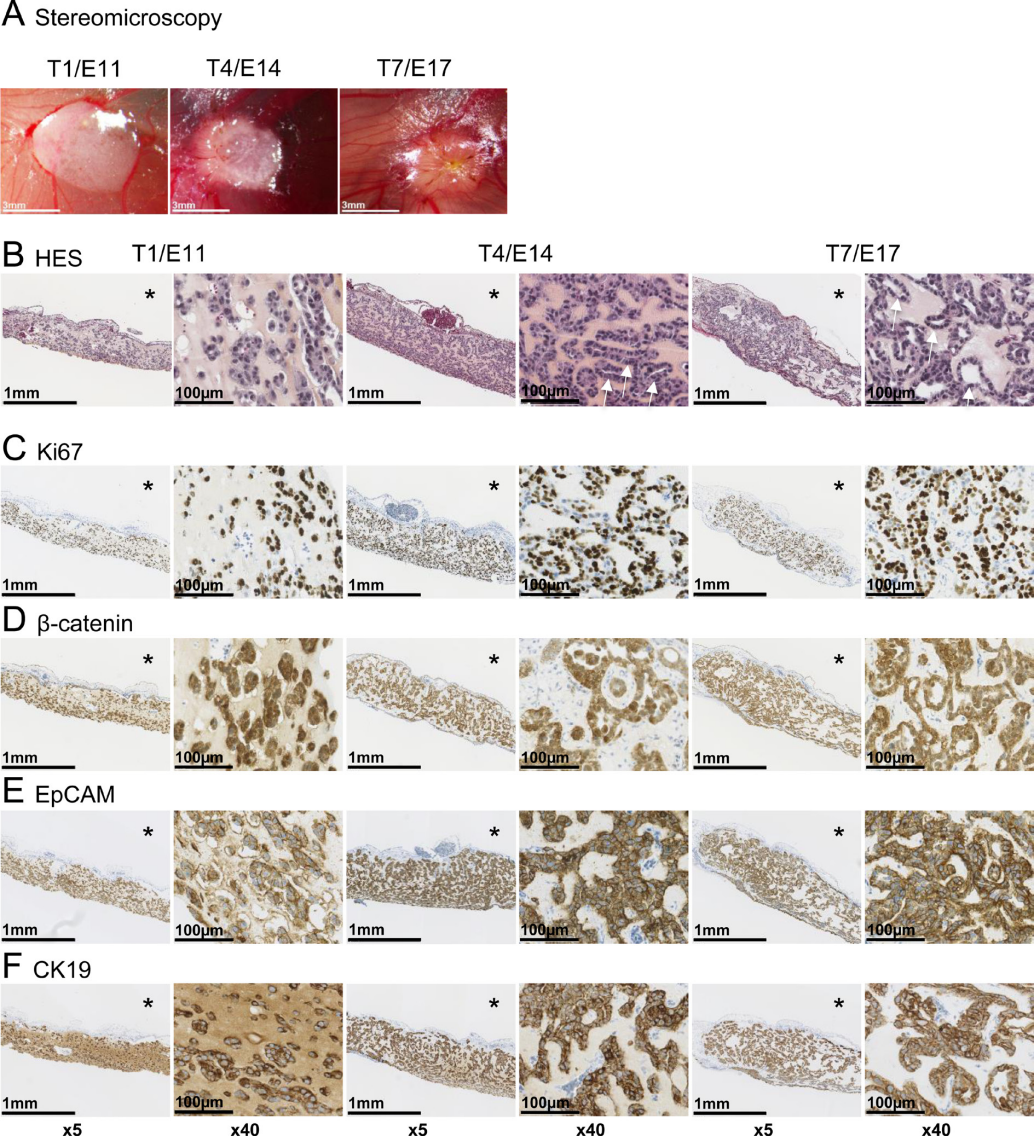
Histological and immunohistochemical analysis of experimental hepatoblastoma on the CAM (**A**) Stereomicroscopy monitoring of Huh6 cell growth and tumor formation on the CAM. (**B**) HES staining of paraffin-embedded tumors from T1 to T7 (arrows: acini-like structures; ^*^ = CAM). (**C**–**F**) Strong immunoreactivity for the proliferation marker Ki-67 and indicated proteins commonly found in hepatoblastoma tissue. Note that no signal was found in the CAM itself (asterisks), except for beta-catenin at the surface of CAM epithelial cells.

At T1/E11 (T = Tumor day, E = embryonic day), a defined area on the CAM was covered by the tumor cell/matrigel mix, which adheres to the CAM surface (Figure 1A). Some bleeding was observed due to CAM laceration prior implantation. Tumors were grown until T7/E17, without clear externally visible changes in morphology except progressive covering of the experimental tumor with CAM cells (Figure 1A, T7/E17). HES staining of paraffin-embedded tumors show unorganized Huh6 cells in matrigel on T1/E11, which subsequently change morphology and organize themselves into acini-like structures visible on T4/E14 and T7/E17 (Figure 1B, arrows). Tumor cell organization was accompanied by continuous cell proliferation as evidenced by strong nuclear expression of Ki67 protein in the majority of tumor cells, clearly visible at T4/E14 and T7/E17 (Figure 1C). Importantly, experimental tumors maintained expression of cytoplasmic and nuclear β-catenin (Figure 1D). EpCAM (CD326), a surface receptor implicated in cell adhesion and associated with poor clinical outcome, has been shown to be expressed in a mouse model of HBL [25]. Strong immunoreactivity for this tumor marker was evidenced in experimental tumors grown on the CAM (Figure 1E). CK19, another protein frequently found overexpressed in HBL cells, was found highly expressed at the membrane of tumor cells on CAM (Figure 1F). For all antibodies used, the CAM itself (asterisks) showed no positive reactivity, except for beta-catenin (Figure 1D), which stained CAM epithelial cells. Taken together, these morphological and immunohistological results suggest that the chicken embryo CAM stroma provides a microenvironment favorable for short-term HBL growth.

### RNA-sequencing, DE genes and bioinformatics results

**Figure 2:**
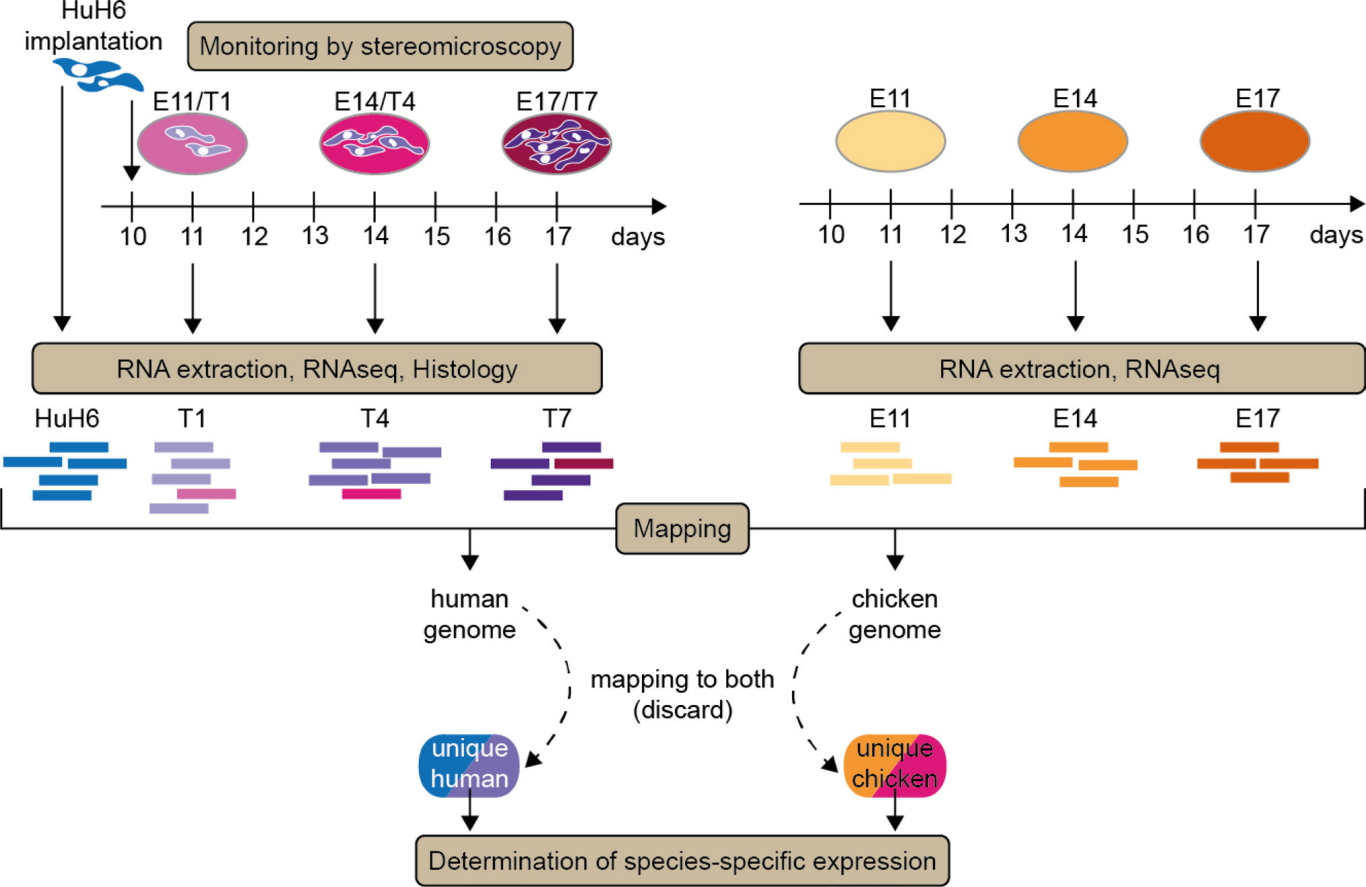
Implantation and sequencing strategy The schema is presenting the overall model and gene selection set-up. Huh6 cells were implanted on embryonic day 10 (E10) and tumors isolated one (T1, E11), four (T4, E14) and seven (T7, E17) days later. At each day, photos were taken and representative tumors isolated for further processing (histology, RNA extraction). Normal CAMs at the same days were also isolated as reference tissue containing only chick genes as well as Huh6 cell mRNA prior to implantation on the CAM. Species-specific gene regulation strategies are applied at indicated stages/tumor progression days.

**Figure 3:**
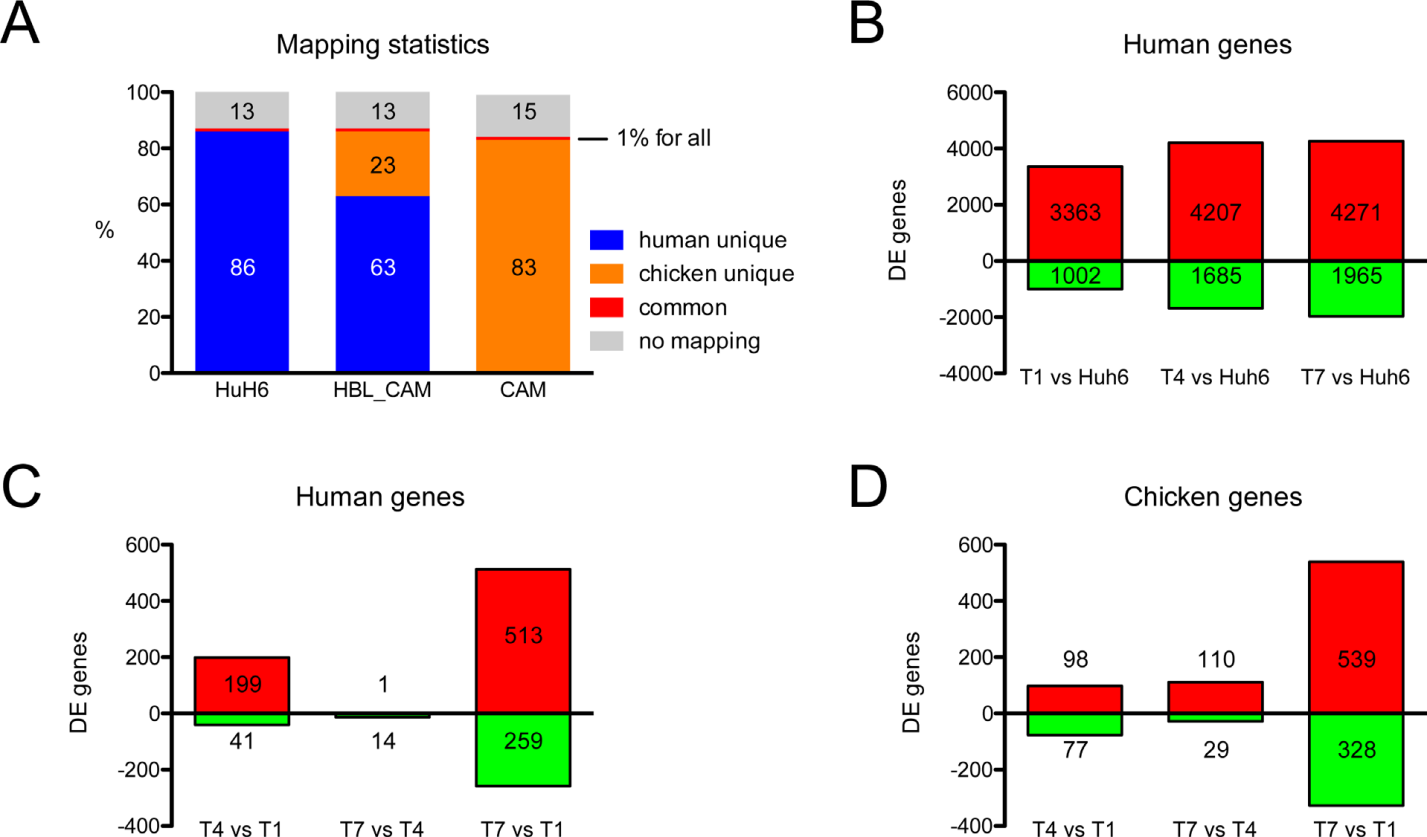
RNA-seq mapping statistics and significantly regulated genes (**A**) Due to experimental design there was a need to separate the origin of RNA reads. Therefore, reads were mapped to both human and chicken genomes. Around 1% of them were mapped to both species in any of the samples, whereas around 14% failed to map. See Results for naming of resulting read subsets. (**B**) A large number of genes were significantly regulated between Huh6 cells *in vitro* and different days on CAM (T1-7) with many genes upregulated. Regulation of genes expressed by Huh6 cells (**C**) and chicken host (**D**) during growth on CAM.

To understand the development of Huh6 cells on the CAM at a molecular level, we performed RNA-seq transcriptional analysis of tumors on days T1, T4 and T7, and in parallel of CAM on days E11, E14 and E17 (Figure 2). Since each tumoral sample grown on CAM contained fraction of chicken cells we used a conservative experimental strategy allowing us to clearly distinguish the origin of each read. We mapped all RNA-seq reads to both genomes and used for subsequent analysis only those uniquely mapping to either chicken or human genome (Figure 3A). This protocol resulted in 86% of reads coming from Huh6 cell RNA mapping only to the human genome, one percent of reads were common to human and chick and 13% failed to map. Similar proportions were found for reads coming from CAM RNA: 83% mapped to chicken genome, 1% was ambiguously mapped and 15% failed to map. In CAMs bearing Huh6 cells 63% of reads matched to human only, 23% to chicken, 1% to both and 13% did not map.

**Figure 4:**
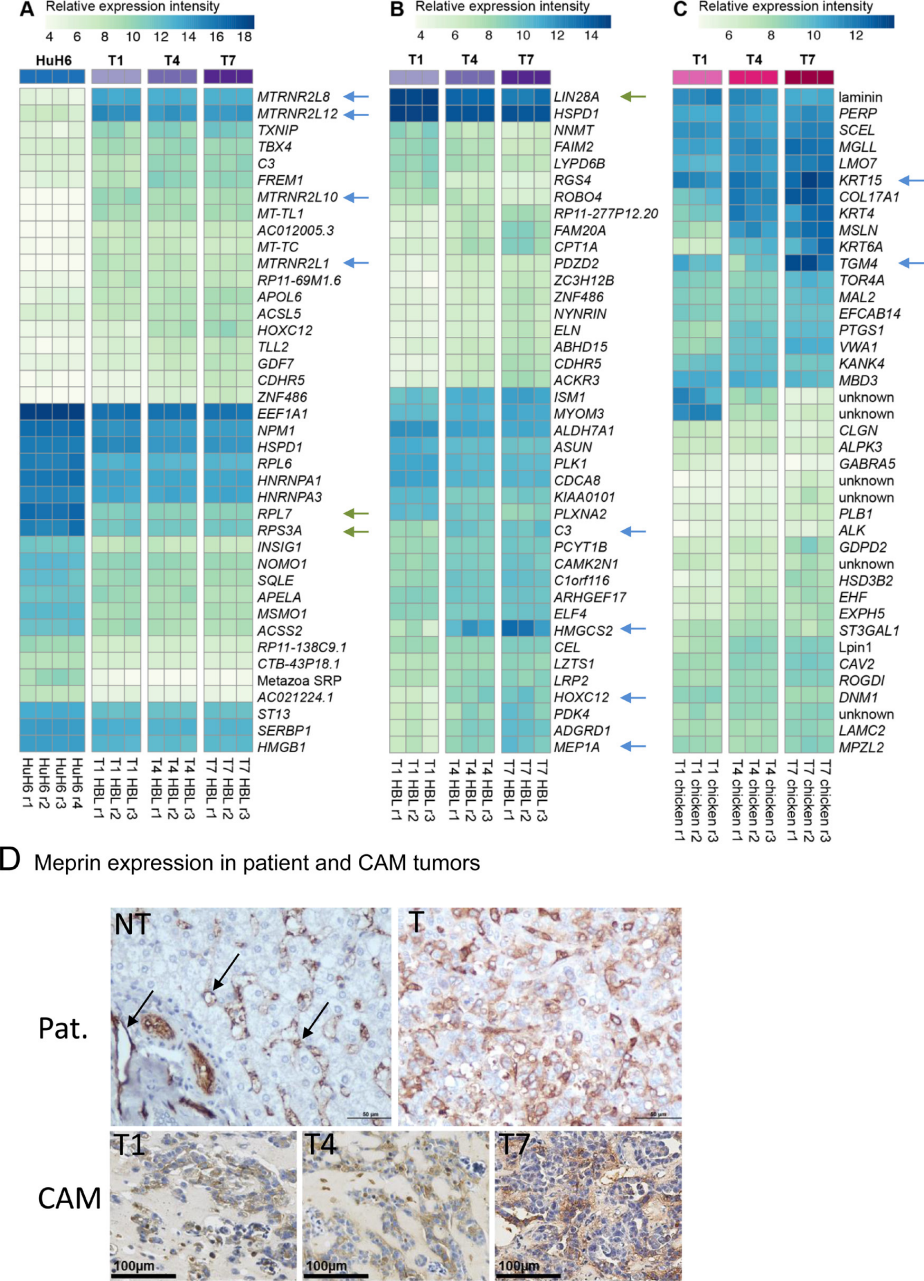
Differentially expressed human and chick genes (**A**) Striking differences in gene regulation were observed between *in vitro* cultured Huh6 cells and CAM-implanted cells. (**B**) Top 40 differentially expressed human genes during short-term evolution of experimental HBL tumors from T1 to T7. Arrows point to genes strongly induced during tumor growth on CAM. (**C**) Chicken genes regulated in the tumor stroma and adjacent CAM. (**A**–**C**) Upregulated and downregulated human genes are indicated with blue and green arrows, respectively. (**D**) Comparison of meprin-1α expression in a representative patient (Pat.) hepatoblastoma with experimental HBL grown on the CAM. Arrows point to the wall of blood capillaries in non-tumoral liver (NT), whereas in HBL, cytoplasmic staining is found in tumor cells, in a similar manner as on the CAM.

We analyzed differentially expressed (DE) genes for each subset of samples. We found that 3,363 human genes are upregulated in the developing Huh6 tumors as compared to the cell line, whereas 1,002 genes are downregulated (Figure 3B). We compared the numbers of DE genes during the course of experiment separately for human genes originating from Huh6-derived tumors (Figure 3C) and for chicken genes in tumor stroma/adjacent CAM (Figure 3D). We noticed that only 15 human genes were regulated between T7 and T4, whereas the chick stroma transcriptome changes gradually with 139 genes differentially expressed in the same time frame. We then analyzed in details the top DE genes between Huh6 cells before and after implantation (Figure 4A), tumor transcripts (Figure 4B) and chicken transcripts during experiment (Figure 4C). The top genes upregulated in implanted Huh6 belong to a family of MT-RNR2-like genes encoding humanin peptides, whereas some of the most downregulated genes are ribosomal proteins *RPL7* and *RPS3A* (Figure 4A and Supplementary Table 1). In general, Huh6 cells growing on CAM start to express some of the typical liver genes including complement component *C3* and many members of the cytochrome p450 family (Supplementary Table 4–6). Ingenuity pathway analysis (IPA) revealed also upregulation of many homeobox proteins including *HOXC12*, *HOXA9* and *HOX* (Supplementary Figure 1). During the course of tumor development we observed a slight but significant decrease of *LIN28A* stem cell marker typical for HBL and an increase of 3-hydroxy-3-methylglutaryl-CoA synthase 2 *HMGCS2* and *MEP1A* (Figure 4B, Supplementary Tables 2 and 7–9). On the other hand, the CAM during the course of the experiments expresses typical genes involved in chicken development like *TGM4*, *KRT15* and *NEU2* (Figure 4C, Supplementary Tables 3 and 10–12). Because we detected an upregulation of *MEP1A*, a gene normally expressed in small intestine and colon, in Huh6 cells grown on the CAM, we set out to confirm the relevance of its expression in HBL and confirmed the overexpression of the protein by immunohistochemistry in a patient sample and in Huh6 tumors grown on CAM (Figure 4D).

**Figure 5:**
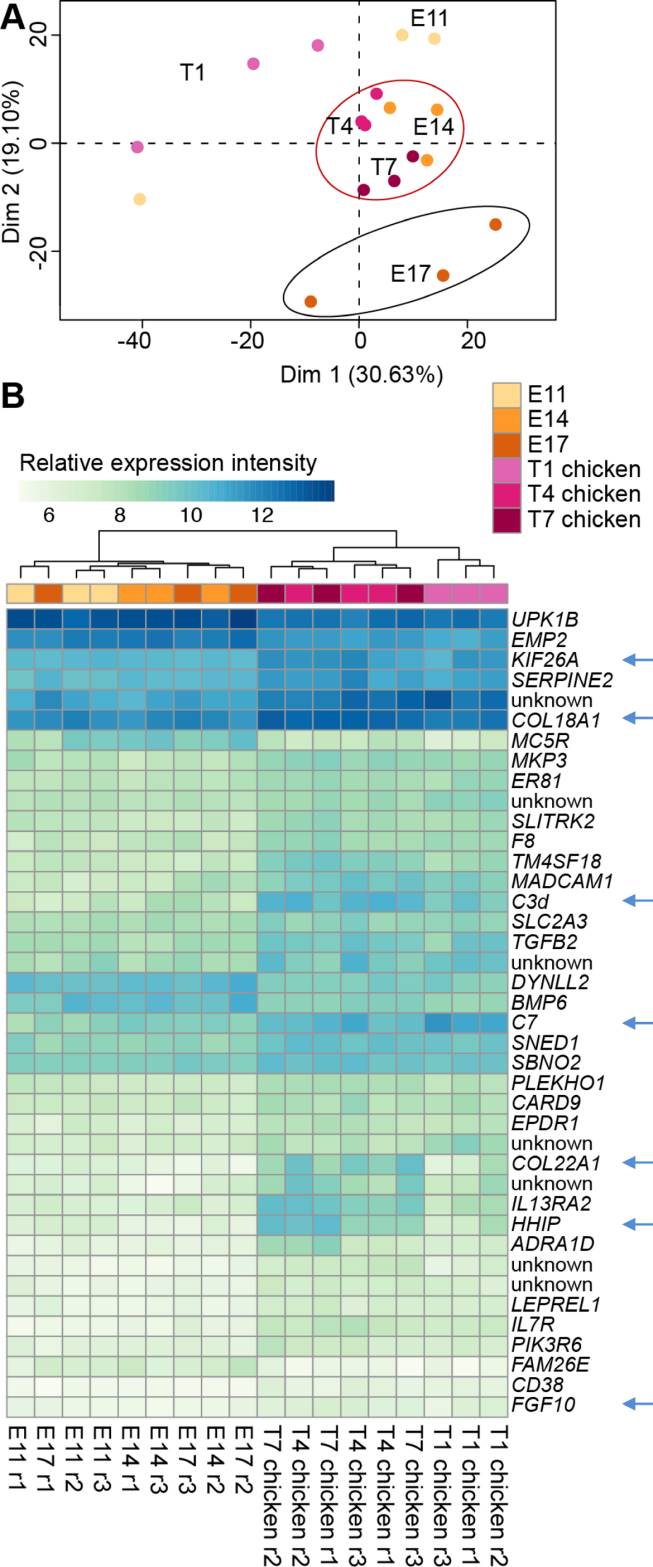
Comparison of CAM transcriptomes during normal development and with Huh6 tumor growth (**A**) Principal component analysis (PCA) of 1,000 most expressed genes in CAM samples. Difference between E17 and E14 is more pronounced than between T7 and T4 despite the same time frame. (**B**) Heatmap of top 40 genes differentially expressed between normal CAMs and corresponding CAMs implanted with tumor cells. Arrows point to interesting candidate genes, whose pro-tumoral functions are discussed in text. Samples on both panels are colored according to the legend. Normal CAM samples are listed as E11, E14 and E17, whereas CAM with Huh6 tumors are listed as T1, T4 and T7 chicken.

Lastly we wanted to focus on the transcriptional differences in the CAM tissue that are specifically induced by the growth of Huh6 tumors. We first performed a principal component analysis of the 1,000 most expressed genes in non-implanted CAM (E11, E14 and E17) and in CAM bearing tumors at the corresponding developmental days (Figure 5A). At day 1, CAMs with (T1) or without (E11) Huh6 tumor cells showed a heterogeneous distribution, most likely reflecting differences in development speed, more pronounced in earlier stages of development. At day 4, as the CAM vasculature becomes mature, differences between Huh6-implanted CAMs and non-tumor-bearing (normal) CAMs were minimal (compare T4 and E14). Finally, at day 7 normal CAMs (E17) were different from tumor CAMs of the same day (T7), and T4 and T7 tumors grouped together with the E14 CAMs indicating a more immature phenotype of E17 CAMs with tumors cells (Figure 5A). Then we looked at the most deregulated genes in CAM with tumors and found genes responsible for proliferation like *KIF26A*, *HHIP* and *FGF10*, collagens *COL22A1* and *COL18A1* and complement components *C7* and *C3d* (Figure 5B and Supplementary Table 13). IPA analysis revealed that many of the deregulated genes in the chick stroma in presence of Huh6 cells are participating in the formation of blood vessels (Supplementary Figure 2). These genes include *VEGFA*, the major proangiogenic growth factor, together with one of its receptors, *FLT1*, but also other proangiogenic genes such as *ANGPT1*, *CXCR4*, *FN1* and *HGF*.

### *In vivo* growth inhibition of experimental tumors by cisplatin

**Figure 6:**
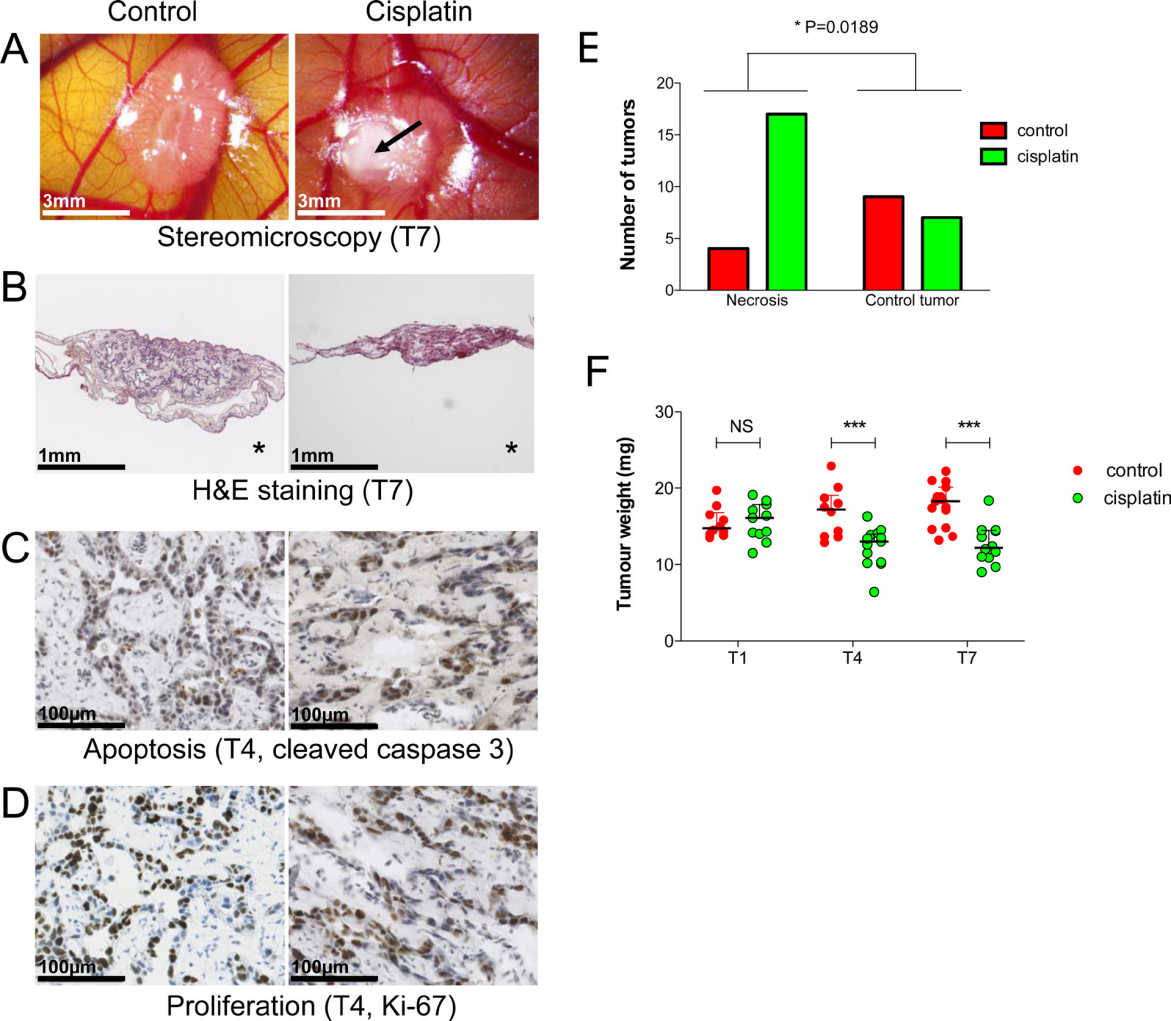
Cisplatin effects on experimental hepatoblastoma on the CAM (**A**) Stereomicroscopy of control and cisplatin-treated tumors at T7 on the CAM. Signs of necrosis (arrow), a phenotype used to evaluate treatment efficacy (see E). (**B**) HES staining confirms tumor growth inhibition after cisplatin treatment. (**C, D**) Immunohistological demonstration of increased apoptosis and decreased cell proliferation in the cisplatin group. (**E**) Quantification of cisplatin effects based on phenotypic examination of tumors (signs of necrosis). (**F**) HBL growth inhibition by cisplatin at T4 and T7 evidenced by tumor weight.

To validate the model for experimental treatments, we applied standard chemotherapy to HBL CAM tumors. Cisplatin treatment leads to significant growth inhibition in the HBL CAM model as evidenced by HES staining and several biological parameters (Figure 6). First, cisplatin-treated tumors showed signs of necrosis on the surface more frequently compared to control tumors (Figure 6A, 6E). No effect of cisplatin treatment on tumor weight is observed at T1, but at T4 and T7, a significant reduction of tumor weight is evidenced (*P* < 0.001, Figure 6F). This is further confirmed by HES histology (Figure 6B), as well as the strong presence of apoptotic cells (cleaved caspase 3 staining; Figure 6C) although treated tumors still contained Ki-67 positive cells (Figure 6D). These results suggest that the model is suitable for testing novel anti-tumor drugs in a controlled *in vivo* environment within one week only.

## DISCUSSION

To model tumor-host interactions *in vivo*, we have established several models of highly aggressive tumors using the chick chorioallantoic membrane as host tissue [16, 19, 26, 27]. Cellular and molecular characterization of these models using microarrays revealed expression of genes playing important roles in human tumors as well. To uncover novel gene deregulations using our model, we focused here on HBL, a rare but aggressive childhood tumor. Although high-throughput gene expression studies have been performed to compare HBL to normal liver, [28] such studies cannot distinguish between tumor genes and stroma-expressed genes. However, it has been shown recently that the stroma compartment reacts specifically to tumor growth, and genes expressed by cancer-associated fibroblasts (CAFs) or other stromal cells can predict clinical outcome to anti-cancer therapy [29]. It is therefore of interest to develop strategies to assign gene deregulation either to the cancer cells or their supporting stroma. Few studies have addressed this question so far. We have shown for the first time that such a species-specific high throughput strategy is feasible *in vivo* using the CAM model and can lead to the discovery of tumor progression genes in cancer cells which are not expressed *in vitro*, such as *NTN1* encoding netrin-1 or the chemokine *CXCL4V1* [19, 20]. Other groups have since demonstrated the usefulness of this approach for the study of brain metastasis [30] and colon carcinoma [29] and Bradfort *et al*. have improved the technique by applying RNA-seq to xenografts in mice to identify tumor and host expression signatures triggered by anti-angiogenic treatment [31]. Valdes *et al*. have undertaken a systematic RNA-seq and microarray study to investigate the degree of cross-hybridization and cross-alignment between mouse and human cell mixes [32]. Only a few percent of the genes cross-reacted. However, some important cancer progression genes were in these lists. In our system, less than 1% of the genes cross-aligned, most likely due to the greater phylogenetic distance of chick and human (320 MYA) compared to human and mouse (90 MYA). It is surprising that from a morphological point of view, experimental tumors on the CAM shared several important features with orthotopically implanted tumors in mice, such as cytokeratin 19 and beta-catenin expression [2, 33]. Only human tumor cells able to communicate with host cells can form tumors on CAM, through secretion of growth-promoting molecules such as growth factors which activate their receptor on chicken cells. This has been evidenced for vascular endothelial growth factor (VEGF) and its receptor KDR [16].

### Chick genes

Increased expression of *COL17A1*, *KRT4*, *KRT6A* and *KRT15* in CAM with tumors (T7 CAMs compared to T1 CAMs) most likely reflect normal differentiation processes. However, higher levels of CAV2 in T7 CAMs could be interpreted as a sign of increased activation of the endothelium due to the presence of cancer cells [34]. Angiogenic activity in the CAM tissue decreases in the course of development, [35] and at E13, the CAM vasculature is quiescent. Compared to normal CAMs, Huh6 tumor CAMs show altered expression of a network of genes regulating basic endothelial cell function such as adhesion and differentiation, as revealed by IPA analysis (Supplementary Figure 2). These include genes such as *HGF*, *FN1*, *CXCR4*, *ANPT1* and *FLT1*, amongst others. This program is most likely triggered by *VEGFA* secreted by Huh6 cells, which display 3.6-fold increase of this gene at T4 and 2.8-fold at T7 compared to *in vitro* culture conditions (Supplementary Tables 5, 6). The more immature nature of tumor-bearing CAMs is also evidenced by PCA analysis, where T7 CAMs group together with E14 CAMs, whereas E17 CAMs are placed clear away from this group (Figure 5). Among the top 40 regulated genes, we also found increased expression of hedgehog-interacting protein (*HHIP)*, which plays important roles in promoting cancer cell growth in the stromal compartment [36].

### Human genes

Interestingly, we found several ribosomal genes such as *RPL6*, *RPL7* and *RPS3A* downregulated in Huh6 cells on the CAM compared to *in vitro* culture. Extra-ribosomal functions of ribosomal proteins have now retained attention since they play important roles in cancer progression [37]. In zebrafish, loss of function of ribosomal proteins leads to growth impairment, but also predisposes to cancer development [38]. *RPL7* and *RPS3A* are highly expressed in Huh6 cells *in vitro*, but become strongly downregulated in tumors on the CAM (Figure 4A). This most likely reflects a profound change in cell behavior in the novel *in vivo* environment, which is also underpinned by the very high number of genes significantly regulated when Huh6 cells interact with the CAM (>6,000). Low levels of some ribosomal proteins might favor tumor growth, since *RPL7A* is strongly downregulated in osteosarcoma tissue compared to normal bone and low levels of *RPL7A* are associated with poor survival [39]. In ovarian cancer, shRNA-mediated knock down of *RPS7* accelerated tumor cell proliferation [40] and silencing of *RPL41*, which is 13.3-fold down-regulated in Huh6 cells on CAM, leads to anchorage-independent growth of fibroblasts and accelerated tumor growth in mice [41].

Some genes strongly induced by tumors on the CAM encode for members of the mitochondrial humanin family (Figure 4A, arrows). *MTRNR2L8* is induced more than 80-fold on the CAM compared to Huh6 cells *in vitro* and is also one of the most abundant genes. Humanins (HNs) are short peptides with strong anti-apoptotic properties. HN specifically interacts with the apoptosis activating protein BAX by preventing its translocation from the cytosol to mitochondria [42] and also inhibits the extra-long form of the proapoptotic protein BimEL [43]. It is tempting to speculate that exposure of HBL cells to the *in vivo* CAM environment triggers an adaptation program which involves reduction of specific ribosomal proteins to favor tumor cell proliferation and increased expression of anti-apoptotic peptides for survival.

Other proteins strongly affected by exposing Huh6 cells to the *in vivo* microenvironment of the CAM belong to the HOX transcription factor family. Members of this family regulate key morphological events during organogenesis and are often deregulated in human cancer progression [44]. The fact that Hox genes such as *HOXD10*, *HOXA9*, *HOXD9*, *HOXD13* are strongly upregulated in Huh6 cells on the CAM reflect a profound reprogramming of cancer cells in a permissive, growth promoting *in vivo* microenvironment (Supplementary Figure 1).

Huh6 cells organize themselves into acini-like structures after several days on the CAM could be regarded as a differentiation process. Interestingly, mitochondrial *HMGCS2*, which encodes for an enzyme of the ketogenic pathway, increases 27-fold from T1 to T7 and its expression controls differentiation in colon and breast cancer cells [45, 46]. *HMGCS2* plays a critical role in tumor progression, since overexpression increases ketone body production, thereby favoring growth and motility of cancer cells [47].

We validated expression of meprin-1α encoded by the *MEP1A* gene, which strongly increases in tumors on the CAM, in a HBL patient tumor by immunohistochemistry. Meprin-1α has not yet been described in this pathology, but strong overexpression in poorly differentiated hepatocellular carcinoma has been reported [48]. In normal liver, meprin-1α is expressed in blood vascular and sinusoidal endothelial cells (Figure 4D), whereas in HBL, additional expression occurs in the cytoplasm of cancer cells. Meprin-1α expression is associated with increased invasion and angiogenesis in colon cancer [49]. Further studies should address the protumoral role of MEP1A in HBL given its strong induction in the model and expression in patient tumors.

Taken together, our morphological and molecular characterization of HBL growth using the CAM model sheds new light on the dynamics of the adaptation potential of tumor cells to the microenvironment. Our model should be useful to validate novel therapeutic strategies for HBL with bad prognosis. It should be emphasized that the CAM model system could now be used to implant freshly isolated patient tumor material including HBL tissue [27, 50]. Probing the growing patient-derived tumor with RNA-seq could identify the molecular landscape of tumor regrowth individually and reveal novel, personnel therapeutic targets. Subsequent treatment of patient-derived tumors on the CAM with adequate therapeutics could personalize pediatric anti-cancer treatment.

## MATERIALS AND METHODS

### Cell culture and cell line authentication

Huh6 cells were received from the Japan Health Sciences Foundation, Osaka, Japan. They were grown at 37° C in a humidified atmosphere of 5% CO_2_ in D-MEM Glutamax, 1 g/L D-glucose (Gibco) supplemented with 10% fetal calf serum (FCS, Sigma), 100 U/ml of penicillin and 100 μg/ml streptomycin (Gibco). The identity of the cells was verified once a year by Short Tandem Repeat (STR) profiling (LGC Standards) and found to be identical to original Huh6 markers.

### Chick embryos and implantation on CAM

Fertilized chick embryos were handled as described [16]. For implantation 1×10^6^ cells were included in 40μL Matrigel^®^ (50% culture medium/50% Matrigel^®^, growth factor reduced, Corning), incubated 45 minutes at 37° C for polymerization and then deposited on the CAM after mild laceration of the membrane. Three to four independent transplantation experiments with at least 10–30 embryos per group were carried out. Experimental tumors were photographed *in vivo* using a Nikon SMZ800N Stereomicroscope, connected to a digital image camera (DS Fi2) piloted by Nikon’s Digital Sight DS-U3 microscope camera controller and processed using Nikon NIS Elements software (version D4 20.00).

### Tumor isolation and RNA extraction

At indicated days tumors or non-implanted CAMs were isolated, washed in PBS and snap frozen in liquid nitrogen. RNA extraction was carried out using the mirVana^TM^ Isolation Kit (Ambion) according to the manufacturer’s protocol. RNAs of Huh6 cells in culture were also extracted with the same kit according to the manufacturer’s protocol. RNA quality was verified using Agilent RNA 6000 Nano kit on an Agilent 2100 Bioanalyzer (Agilent Technologies).

### RNA sequencing and analysis

Four μg of RNA were used to generate sequencing library using the TruSeq Stranded mRNA Sample Preparation kit (Illumina) according to the standard protocol. Library preparation and sequencing were performed by the MGX-Montpellier GenomiX platform. We sequenced three biological replicates of CAMs and Huh6 deposited on CAM on three time points: day 11, 14 and 17, named E11, E14, E17 (embryonic development day - CAM) and T1, T4, T7 (tumor day - Huh6), see Figure 1. Thus altogether 18 samples were sequenced on HiSeq 2500 (Illumina) using three lanes of a flow cell resulting in on average 40 million 50-nt single-end raw reads per sample. Additionally, four Huh6 RNA samples were sequenced on HiSeq 2500 (Illumina) using half of a flow cell lane, resulting in on average 34 million paired-end raw reads per sample (named Huh6 r1-4), For each sample all resulting reads were mapped to both human (GR38) and chicken (GalGal4) genomes plus transcripts by hisat2 (2.0.3-beta) with default settings [21]. We excluded reads or read pairs that simultaneously mapped to both genomes and kept for subsequent analysis only those that mapped uniquely to either human or chicken genome. Reads were summed-up at the gene level (Ensembl 82) by *feature Counts* [22]. Differential gene expression analysis was performed by *DESeq2* [23]. We included all genes with adjusted *P*-value < 0.05 and absolute value of log2-fold change >1 (Figure 3, Supplementary Tables 4–12). All heat maps were generated by plotting the number of reads with variance stabilizing transformation (*DESeq2*) using *heatmap.2* function of the R package and *gplots* or *pheatmap* using Euclidian distance and complete clustering method. Differential gene expression results from *DESeq2* were also analyzed using QIAGEN’s Ingenuity^®^ Pathway Analysis (IPA^®^, QIAGEN Redwood City, www.qiagen.com/ingenuity), focusing on upstream transcriptional regulators and affected signaling networks.

The datasets generated and/or analyzed during the current study are available at the Gene Expression Omnibus repository, (https://www.ncbi.nlm.nih.gov/geo/) under the number GSE101413.

### Histology and immunohistology

One day (T1), four days (T4) and seven days (T7) after the implantation of the cells (Figure 1), the CAM containing the tumors were fixed *in situ* for 1 hour with formalin (Diapath). In parallel, non-implanted CAMs of corresponding developmental days were fixed using the same protocol. After fixation CAMs were cut out, washed in PBS and stored in 70% ethanol. Huh6 cells were washed with PBS, fixed with formalin (Diapath), suspended in 1% agarose and stored in 70% ethanol. CAMs and cells were then embedded in paraffin and cut into 5 μm sections using a microtome. Hematoxylin/eosin/safran (HES) staining and processing of slides prior to immunostaining was performed using standard procedures. The following primary antibodies were used: anti-β-catenin (1:5000, BD Biosciences, Clone 14, 610154), anti-EpCAM (1:250, Dako, Clone Ber-EP-4, M0804), anti-CK19 (1:50, Dako, Clone RCK108, M0888), anti-Ki67 (1:100, Dako, Clone MIB-1, M7240), anti-Caspase 3 activated (1:200, R&D Systems, AF835) and anti-Meprin-1α (1:100, R&D Systems, AF3220). HRP-coupled secondary antibodies were from the EnVision^TM^ FLEX kit (Dako) and for anti-Meprin1A, an anti-goat antibody (HRP ImPRESS^TM^ anti-goat IgG, Vector Laboratories) was used. Images were taken using a Nanozoomer PLC (Hamamatsu) at the Bordeaux Imaging Center (BIC http://www.bic.u-bordeaux.fr).

### Experimental tumor treatment and phenotypic quantification

Cisplatin (Sigma) was lyophilized and suspended in 0.9% saline solution. The drug was added to the cells together with the Matrigel^®^ to a final concentration of 15 μM, prior to implantation on the CAM. Tumor growth was monitored daily and experiments terminated at E17/T7. Tumors were isolated and weighted at T1, T4 and T7 and tumor bearing CAMs were isolated for histology at T1, T4 and T7. A total of 35 control and 36 cisplatin-treated tumors have been analyzed, embryos that died during incubation were excluded. To evaluate biological effects of cisplatin treatment, a semi-quantitative approach was used, adapted from Auf *et al*. [24]. Stereomicroscopy photos of all treated and control tumors were randomized using a randomizer script generated by the Script Editor of Apple MacOS El Capitain system. Randomized photos were presented to three observers familiar with the model but not implicated in the design of this study. Observers noted yes (=1) if presence of white areas on the tumor or not (=0) and if tumors resembled to a normal tumor (=1) or not (=0). Data were only processed for tumors where two out of three observers concluded the same result. A contingency table was generated and analyzed using the Chi-square test (GraphPad Prism 5, GraphPad Software).

## SUPPLEMENTARY MATERIALS FIGURES AND TABLES







## Abbreviations

CAM: chick chorioallantoic membrane
DE: differentially expressed
HBL: hepatoblastoma
RNA-seq: RNA sequencing
