# Supplementary material for: Tracking cellular and molecular changes in a species-specific manner during experimental tumor progression in vivo
Source: Oncotarget. 2018 Mar 1;9(22):16149–62. doi: 10.18632/oncotarget.24598 (PMC5882324; doi:10.18632/oncotarget.24598)
Supplement: Supplementary file 1 [file oncotarget-09-16149-s001.pdf]

Tracking cellular and molecular changes in a species-specific manner during experimental tumor progression *in vivo*

SUPPLEMENTARY MATERIALS

A IPA analysis of Huh6 cells in vitro vs T7 Huh6 tumors on CAM

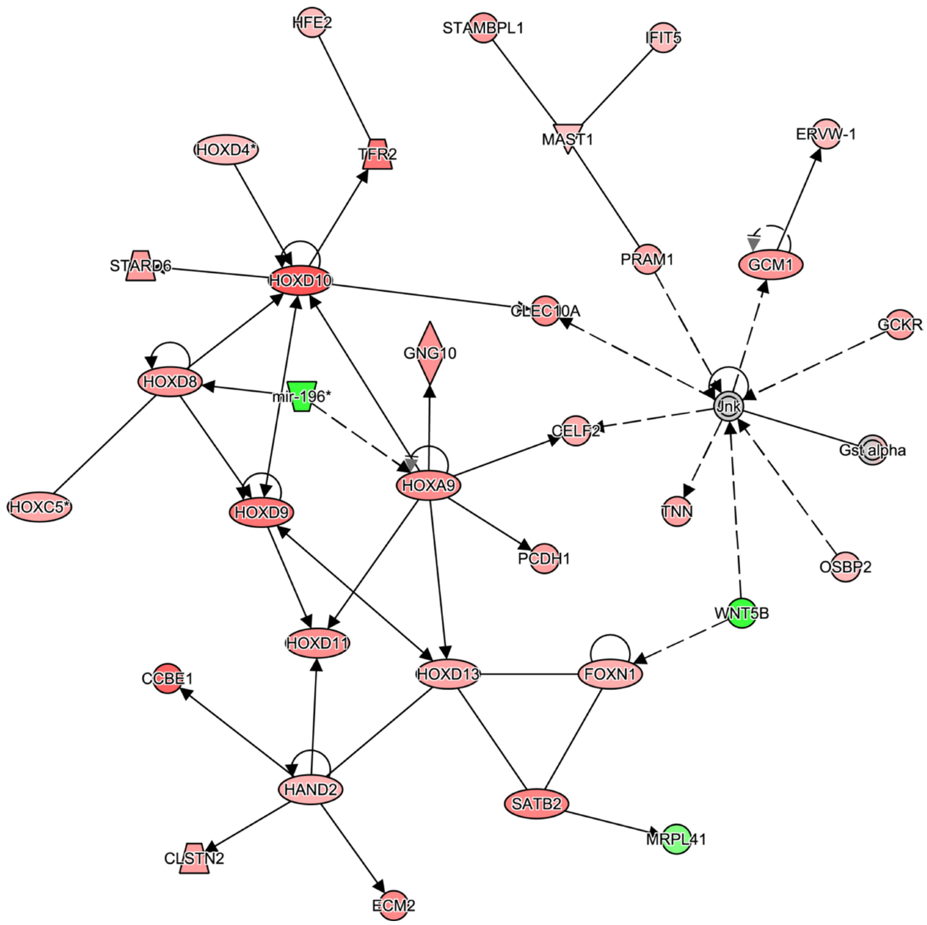

Supplementary Figure 1: IPA analysis gene networks differentially expressed between *Huh6 in vitro* and T7 tumors on CAM. IPA analysis with 1,000 genes input revealed interaction of a large number of HOX genes.

A IPA analysis of normal CAM vs Huh6-implanted CAMs at T7

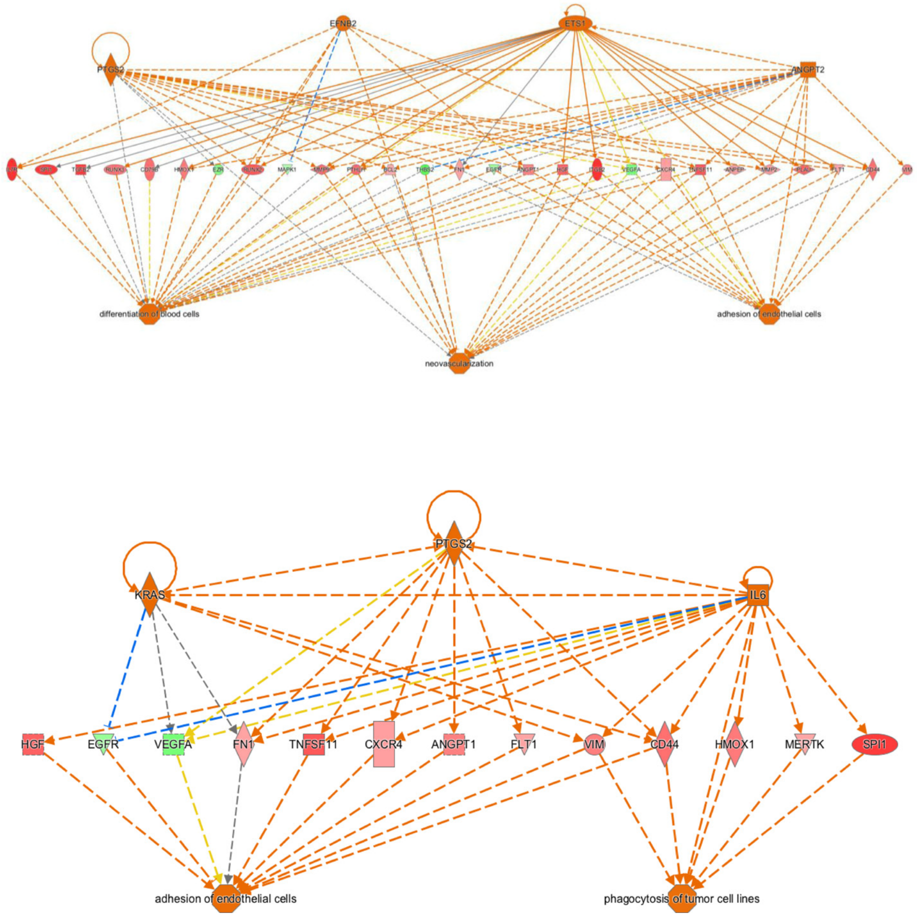

Supplementary Figure 2: IPA analysis gene networks differentially expressed between normal CAMs and T7 CAM. IPA analysis with 1,000 genes input revealed modification of the CAM stroma with increased expression of genes favoring endothelial cell adhesion and angiogenesis.

Supplementary Table 1:Top 20 genes based on fold-change between Huh6 *in vitro* and T7 CAMs

Gene symbol	baseMean	Fold-Change (log2)	*p*-value (adjusted)	Description
**MTRNR2L8**	**5793.16**	**8.35**	**6E–152**	**MT-RNR2-like 8**
**MTRNR2L10**	**409.97**	**8.27**	**3E–79**	**MT-RNR2-like 10**
**MTRNR2L1**	**161.26**	**7.24**	**4E–52**	**MT-RNR2-like 1**
**MTRNR2L12**	**13925.83**	**6.82**	**1E–96**	**MT-RNR2-like 12**
AC012005.3	97.73	6.73	8E–47	Uncharacterized protein
ZNF486	38.61	6.55	3E–34	zinc finger protein 486
CDHR5	54.63	6.54	3E–40	cadherin-related family member 5
PGLYRP2	79.81	6.23	6E–24	peptidoglycan recognition protein 2
HOXC12	270.13	6.18	9E–46	homeobox C12
TLL2	100.71	5.95	1E–41	tolloid-like 2
ACSS1	20.12	5.93	5E–25	acyl-CoA synthetase short-chain family member 1
HMGCS2	2292.04	5.89	2E–27	3-hydroxy-3-methylglutaryl-CoA synthase 2 (mitochondrial)
TXNIP	1040.58	5.87	3E–34	thioredoxin interacting protein
CLIC6	52.70	5.78	2E–27	chloride intracellular channel 6
HSD17B2	150.79	5.59	1E–26	hydroxysteroid (17-beta) dehydrogenase 2
TRIM31	22.54	5.35	4E–25	tripartite motif containing 31
GOLGA8R	29.54	5.33	2E–26	golgin A8 family, member R
TNFSF15	15.96	5.24	7E–18	tumor necrosis factor (ligand) superfamily, member 15
PDK4	259.59	5.18	4E–27	pyruvate dehydrogenase kinase, isozyme 4
AC005480.1	26.72	5.15	5E–22	Uncharacterized protein
**RPL7**	**26226.20**	**–5.84**	**3E–159**	**ribosomal protein L7**
PLA2G3	79.01	–5.11	7E–21	phospholipase A2, group III
PCSK9	409.49	–5.03	2E–30	proprotein convertase subtilisin/kexin type 9
NMRK2	124.63	–4.40	5E–13	nicotinamide riboside kinase 2
CYP1A1	87.49	–4.17	3E–21	cytochrome P450, family 1, subfamily A, polypeptide 1
INSIG1	894.83	–4.05	2E–42	insulin induced gene 1
**RPS3A**	**15895.53**	**–3.93**	**1E–35**	**ribosomal protein S3A**
GOLGA8M	11.29	–3.80	2E–07	golgin A8 family, member M
**RPL41**	**283.27**	**–3.74**	**4E–27**	**Ribosomal protein L41, isoform CRA_a**
CTAGE4	15.72	–3.69	8E–11	CTAGE family, member 4
TRIM63	14.87	–3.66	1E–09	tripartite motif containing 63, E3 ubiquitin protein ligase
**RPL18A**	**5496.87**	**–3.61**	**1E–14**	**ribosomal protein L18a**
CAPN14	176.63	–3.50	2E–18	calpain 14
MSMO1	1874.62	–3.49	2E–67	methylsterol monooxygenase 1
PMEL	367.38	–3.49	6E–25	premelanosome protein
UBE2S	309.58	–3.48	6E–13	ubiquitin-conjugating enzyme E2S
AC241377.2	10.60	–3.47	3E–07	Protein LOC100996720
HIST1H4J	7.63	–3.47	7E–07	histone cluster 1, H4j
S100A4	1014.65	–3.45	7E–16	S100 calcium binding protein A4
FDFT1	3025.74	–3.41	7E–28	farnesyl-diphosphate farnesyltransferase 1

Supplementary Table 2: Top 20 genes based on fold-change between T1 and T7 CAMs: human genes

Gene symbol	baseMean	Fold-Change (log2)	*p*-value (adjusted)	Description
**HMGCS2**	**2292.04**	**4.75**	**8E–15**	**3-hydroxy-3-methylglutaryl-CoA synthase 2 (mitochondrial)**
CDHR5	54.63	4.40	7E–18	cadherin-related family member 5
FAM20A	121.47	4.28	1E–16	family with sequence similarity 20, member A
**HOXC12**	**270.13**	**3.89**	**1E–15**	**homeobox C12**
ACKR3	64.08	3.65	8E–15	atypical chemokine receptor 3
DACT2	26.17	3.59	2E–07	dishevelled-binding antagonist of beta-catenin 2
ZC3H12B	30.23	3.49	1E–08	zinc finger CCCH-type containing 12B
ZNF486	38.61	3.40	5E–11	zinc finger protein 486
SLC22A7	12.79	3.39	2E–06	solute carrier family 22 (organic anion transporter), member 7
ADGRD1	341.90	3.38	3E–14	adhesion G protein-coupled receptor D1
CPT1A	143.41	3.31	1E–08	carnitine palmitoyltransferase 1A (liver)
PGLYRP2	79.81	3.30	3E–06	peptidoglycan recognition protein 2
CUBN	1284.97	3.22	2E–07	cubilin (intrinsic factor-cobalamin receptor)
ELN	31.64	3.15	1E–07	elastin
PDK4	259.59	3.15	4E–08	pyruvate dehydrogenase kinase, isozyme 4
**MEP1A**	**328.23**	**3.15**	**9E–10**	**meprin A, alpha (PABA peptide hydrolase)**
NAT8	64.82	3.14	2E–07	N-acetyltransferase 8 (GCN5-related, putative)
HSD17B2	150.79	3.09	6E–07	hydroxysteroid (17-beta) dehydrogenase 2
APOA4	77.24	3.06	3E–06	apolipoprotein A-IV
SLC10A1	13.72	3.04	3E–05	solute carrier family 10 (sodium/bile acid cotransporter), member 1
RGS4	86.60	–3.58	5E–10	regulator of G-protein signaling 4
KIAA0040	20.55	–3.09	9E–07	KIAA0040
S100A9	11.99	–3.05	9E–05	S100 calcium binding protein A9
NNMT	171.24	–3.00	3E–12	nicotinamide N-methyltransferase
ALDH3A1	14.31	–2.80	9E–05	aldehyde dehydrogenase 3 family, member A1
VGF	46.10	–2.75	2E–05	VGF nerve growth factor inducible
DKK4	153.67	–2.69	2E–04	dickkopf WNT signaling pathway inhibitor 4
PPP1R1B	40.95	–2.68	2E–05	protein phosphatase 1, regulatory (inhibitor) subunit 1B
MIXL1	120.82	–2.63	2E–07	Mix paired-like homeobox
ROBO4	78.68	–2.26	8E–08	roundabout guidance receptor 4
PCSK9	409.49	–2.25	5E–05	proprotein convertase subtilisin/kexin type 9
SPTSSB	24.81	–2.16	2E–04	serine palmitoyltransferase, small subunit B
SLC18B1	18.03	–2.10	5E–03	solute carrier family 18, subfamily B, member 1
FAIM2	214.26	–2.09	5E–09	Fas apoptotic inhibitory molecule 2
LAT2	10.72	–2.08	9E–03	linker for activation of T cells family, member 2
PAGE4	1834.52	–2.08	2E–05	P antigen family, member 4 (prostate associated)
EVPL	268.39	–2.08	2E–04	envoplakin
TCAF2	7.39	–2.04	2E–02	TRPM8 channel-associated factor 2
FKBP6	11.63	–2.04	2E–02	FK506 binding protein 6, 36kDa
CD79B	20.64	–2.01	7E–03	CD79b molecule, immunoglobulin-associated beta

Supplementary Table 3: Top 20 genes based on fold-change between T1 and T7 CAMs: chick genes

Gene symbol	baseMean	Fold-Change (log2)	*p*-value (adjusted)	Description
**KRT4**	**2323.93**	**4.95**	**9E–32**	**keratin 4, type II**
HSD3B2	229.34	4.94	1E–37	hydroxy-delta-5-steroid dehydrogenase, 3 beta- and steroid delta-isomerase 2 (HSD3B
TGM4	3832.42	4.65	1E–25	protein-glutamine gamma-glutamyltransferase 4
MSLN	2293.93	4.64	2E–39	mesothelin
**KRT6A**	**1094.26**	**4.50**	**4E–22**	**keratin 6A**
COL17A1	6017.75	4.36	3E–36	collagen, type XVII, alpha 1
**KRT15**	**6023.03**	**4.01**	**3E–29**	**keratin, type I cytoskeletal 15**
SPINK5	315.12	3.90	1E–15	serine peptidase inhibitor, Kazal type 5
MYO15L	70.47	3.72	1E–15	Uncharacterized protein
ALK	33.49	3.70	1E–17	anaplastic lymphoma receptor tyrosine kinase
LOC100858007	32.29	3.67	5E–22	Uncharacterized protein
LAMC2	264.60	3.63	3E–33	laminin subunit gamma 2
GDPD2	144.05	3.63	1E–23	glycerophosphodiester phosphodiesterase domain containing 2
EHF	104.71	3.46	6E–17	ETS homologous factor
	61.35	3.45	3E–19	cytochrome P-450 2C45
ELOVL3	83.90	3.39	2E–15	Elongation of very long chain fatty acids protein
LOC425662	63.61	3.37	3E–13	Uncharacterized protein
CHL1	46.11	3.34	5E–12	cell adhesion molecule L1-like
TACSTD2	29.71	3.20	3E–11	tumor-associated calcium signal transducer 2 precursor
TMPRSS11F	329.84	3.13	7E–11	
LOC420770	637.68	–4.47	1E–21	Uncharacterized protein
	30.94	–3.75	6E–15	Uncharacterized protein
GABRA5	23.27	–3.61	3E–16	gamma-aminobutyric acid (GABA) A receptor, alpha 5
**HES5**	**36.90**	**–3.60**	**1E–15**	**transcription factor HES-5**
A2M	695.86	–3.34	2E–21	Uncharacterized protein
HBE	658.45	–3.24	3E–11	hemoglobin subunit epsilon
	262.42	–3.17	4E–11	Uncharacterized protein
CCDC64	18.50	–3.11	1E–11	coiled-coil domain containing 64
LOC419390	46.43	–3.05	4E–14	hairy and enhancer of split 5-like
FIBIN	327.63	–2.97	6E–11	fin bud initiation factor homolog precursor
Gga.11762	16.65	–2.72	1E–07	Uncharacterized protein
SGIP1	14.53	–2.71	3E–09	SH3-domain GRB2-like (endophilin) interacting protein 1
LOC422307	11.64	–2.70	2E–07	Uncharacterized protein
SLC26A4	29.69	–2.60	8E–07	solute carrier family 26 (anion exchanger), member 4
CDH8	7.42	–2.56	2E–07	cadherin-8
	83.36	–2.53	2E–09	Uncharacterized protein
CHRNA7	19.42	–2.52	1E–06	neuronal acetylcholine receptor subunit alpha-7 precursor
LAMA1	2218.78	–2.52	3E–16	laminin subunit alpha-1 precursor
KLHL1	6.91	–2.45	1E–06	kelch like family member 1
LOC422305	436.45	–2.45	2E–06	Uncharacterized protein

Supplementary Tables 4–12: Complete lists of DE genes presented in Figure 3. See Supplementary_Tables_4–12

Supplementary Table 13: Complete list of DE genes presented in Figure 5. See Supplementary_Table_13
